# A Systematic Review of Autobiographical Memory and Mental Health Research on Refugees and Asylum Seekers

**DOI:** 10.3389/fpsyt.2021.658700

**Published:** 2021-06-04

**Authors:** Sanjida Khan, Sara K. Kuhn, Shamsul Haque

**Affiliations:** ^1^Department of Psychology, Jeffrey Cheah School of Medicine and Health Sciences, Monash University Malaysia, Subang Jaya, Malaysia; ^2^Department of Psychology, University of North Dakota, Grand Forks, ND, United States

**Keywords:** refugee, asylum seekers, autobiographical memory, mental health, PRISMA statement, systematic review

## Abstract

Research examining trauma, memory, and mental health among refugee and asylum-seeking people has increased in recent years. We systematically reviewed empirical work focusing on the link between autobiographical memory and mental health among these populations. The review protocol was registered with PROSPERO (CRD42018095888). Six major databases were searched in August-2020 with no time limit for publication. Following PRISMA Statement guidelines, 22 articles reporting ten quantitative, nine qualitative, and three mixed-method studies were selected from 254 articles identified in the initial search. A basic convergent and qualitative meta-integration technique was employed for data extraction. Four recurrent themes were extracted: (1) memory activation method, (2) memory features, (3) memory content, and (4) refugee mental health. Theme 1 illustrates that narrative interviews, important event recall, and cue word methods were used in most studies. Theme 2 highlights that memories of refugee people were often less specific, inconsistent, and negative-focused. Retrieval failure was also common among these people. Theme 3 reveals that refugee and asylum-seeking people frequently discussed their abandoned identities, lost resources, injustices, ongoing sufferings, and pointless futures. Finally, theme 4 identifies the prevalence of various mental health conditions like Post-traumatic Stress Disorder, depression, helplessness, and anger among these people. The results are discussed in the context of the current autobiographical memory and mental health theories, considering refugee-specific experiences in the asylum process and refugee status.

## Introduction

Every year, mass conflict and violence affect a large proportion of the world's population (1, 2). As of 2019, it is estimated there are 79.5 million people worldwide who were forcibly migrated, 26 million of whom were declared to have refugee status (3). Refugee refers to people who fled their own country to escape from war, conflict, and persecution (4). A large segment of refugee people (above 80%) has resettled in camps in either lower or lower-middle-income countries that are generally densely populated (5). Their post-migration life starts with full of challenges in insecure and unstable conditions as recipient countries are often not prepared to accommodate an influx of many people at one time (5). This unpreparedness creates a significant humanitarian crisis. Since forced migration is associated with multiple traumatic life experiences and a great disruption in personal and social life (6, 7), refugee peoples' cognitive functions [e.g., memory; (8, 9)] and mental health are impacted (10–13).

In this systematic review, we summarize the results of autobiographical memory and mental health research that was conducted on refugee and asylum-seeking people resettled in various parts of the world. We have not found any recent comprehensive review on this topic except for a review on adolescent refugee peoples' autobiographical memory (14), although empirical research in this domain has been steadily growing. Psychologically, living in a refugee camp for a protracted period often causes damage to one's self-control, resilience, and hope (15). A previous review on studies involving Western samples revealed that refugee people were at high risk of negative psychological outcomes (16). People exposed to three or more categories of trauma had eight times the risk of mental illness (13). A study on adult refugee people illustrated that those with Post-traumatic Stress Disorder (PTSD) tended to report more information about their traumatic events, even after three years than those who were exposed to trauma but did not have PTSD (17).

### Autobiographical Memory Impairment and Mental Health Outcomes

The link between psychopathology and autobiographical memory impairment is well documented (18). Autobiographical memories are self-reflective, containing sensory-perceptual details of an event; reflecting the rememberer's current goals, emotions, and expectations (19). From a young age, people develop knowledge about themselves, personal judgment, and even personality characteristics from various life experiences and social interactions (20). The ability to recall autobiographical memory assists in developing self-identity and making adaptations to the existing world; guiding the future (19–21). Numerous studies have shown that people with PTSD and depression have impaired autobiographical memory; more specifically, they tend to recall more overgeneral/incomplete memories (OGMs) compared to healthy controls (18, 22–24).

OGM (i.e., lack of specificity in autobiographical memory) is associated with traumatic life experiences (18). Research has shown that people who have had traumatic experiences, for example, a history of abuse, face more difficulty in remembering specific autobiographical memories than participants who do not have such experiences (25). Several studies have established that OGM contributes to the development and maintenance of emotional disorders among trauma survivors (18), such as veterans (23, 26) and people with histories of abuse (27). In other words, emotional disorders may lead a person's memories toward bias in selecting negative and frightening events (28). Similar to adults, trauma-exposed older adolescents reported overgeneral accounts while recalling distressing memories (14). However, the narratives of young adolescents exhibited a dearth of time and place orientation. They tended to describe the facts rather than the emotional content of the events with self-interpretation (29).

According to Conway and Pleydell-Pearce (19), autobiographical memories are constructed by sampling knowledge from the hierarchically organized autobiographical knowledge structure. At the highest layer of the hierarchy lies the most abstract life story schemas along with lifetime period knowledge. At the bottom are highly specific event memories. The construction of an autobiographical memory results from the interaction between the rememberer's working self, conceptual self, and the autobiographical knowledge structure (21). While the working self represents the rememberer's current goals and emotions, the conceptual self contains the preferences and ideology that were gained through schooling and tradition. Research shows that the construction of an autobiographical memory could be disrupted due to inappropriate memory encoding, impaired cognitive processing, or functional avoidance (30). Emotional disorders themselves can manipulate the nature of the memory retrieved (28). For example, anxious people are predisposed to apprehension, and depression makes people biased toward negativity (31). Ultimately, these tendencies can interrupt proper searching and thus, create a barrier to reaching specific event memories (31, 32). Additionally, Rubin et al. (33) suggested that after trauma exposure, a constructive process contributes to the encoding of the traumatic experiences and later influences the retrieval of those memories. This constructive process also affects the retrieval of non-traumatic memories (18, 24). Impaired autobiographical memory after trauma exposure may work as a protective factor against immediate adverse effects from traumatic events (34). Less specific memories can serve as a defensive coping style, which may reduce immediate emotional distress. However, long-term, it can act as a contributing factor to various other mental health problems (18).

### The Current Systematic Review

A significant amount of work has been published on refugee mental health, which has led to the development of new theories and intervention programs (5). Several literature reviews have also been published on mental health outcomes among both young (35–39) and adult refugee people (40–44). A recent systematic review reported the mental health status of Rohingya refugee people and the language and service-related challenges they face in camps in Bangladesh (45). Several reviews also focus exclusively on autobiographical memories of displaced people, such as two recently published reviews on memories of unaccompanied refugee children (14) and adult asylum seekers' subjective experiences of their migration journeys and resettlement processes (46). There also exists a review on the accuracy of autobiographical memories of asylum seekers (47).

However, we noticed a lack of systematic reviews of research investigating the link between autobiographical memory and mental health among refugee and asylum-seeking people. We also found that there are only a few reviews (45, 46) that followed the Preferred Reporting Items for Systematic Reviews and Meta-Analyses (PRISMA) Statement guidelines. The current review is an attempt to fill these gaps. It is important to examine the feature and content of the memories of refugee people as their memories are crucial for establishing their asylum-seeking credibility and global policymaking (8). Retrieving distressful memories, sometimes repeatedly for legal purposes, may cause psychological problems among refugee populations that would require proper therapeutic attention (48). Research has also illustrated that collective memories of refugee people have at least partially influenced their interpersonal bonding, promoted their culture, religion, and language, and indirectly helped improve their new settlement conditions (49).

Besides mental health, this systematic review focused on autobiographical memory rather than other higher-order cognitive processes as that would widen the scope too much. We also included those articles that are written in English because the current authors have proficiency in that language. We aimed to map currently available findings of autobiographical memory and mental health among refugee and asylum-seeking people recruited from different parts of the world. We pose three broad research questions: (1) what are common methods used in refugee and asylum-seeking people's autobiographical memory and mental health research; (2) what are the main features and content of the target population's autobiographical memory, and (3) what kind of mental health outcomes are reported among those populations?

## Methods

### Protocol and Registration

The protocol of this systematic review was registered with PROSPERO (registration number: CRD42018095888).

### Eligibility Criteria

This review included articles that focused on autobiographical memory and mental health issues among refugee and asylum-seeking people. There were three inclusion criteria: (1) any articles examining trauma narratives, autobiographical memory, and life stories; (2) studies that assessed mental health issues using standardized scales, questionnaires, and narratives; (3) only peer-reviewed, scientific articles published in the English language. There were five exclusion criteria: (1) studies focusing on autobiographical memories of displaced individuals within their own country, and war trauma victims who were not refugees or asylum-seekers; (2) studies with a sample of migrated people for immigration purposes, asylum seekers but not a refugee; (3) studies looking at theoretical issues of autobiographical memory; (4) studies administering intervention programs to address mental health and memory-related issues; and (5) studies focusing on other higher-order cognitive processes among refugee people.

### Sources of Information

Before initiating database searching, relevant synonyms for topical keywords (i.e., refugee, autobiographical memory, and psychological outcome) were identified through Medical Subject Headings (MeSH). Search strings included keywords and synonyms connected with Boolean operators (i.e., AND, OR) and advanced search techniques (primarily asterisks for truncation of root words to allow for alternate word endings). An extensive search was then conducted in six major databases: Scopus, PubMed, Web of Science, PsycINFO, PILOTS, and EBSCOhost. The Supplementary Table 1 shows the results from each database using the specified search string. This literature search was conducted in August 2020.

### Article Selection

**Figure 1:**
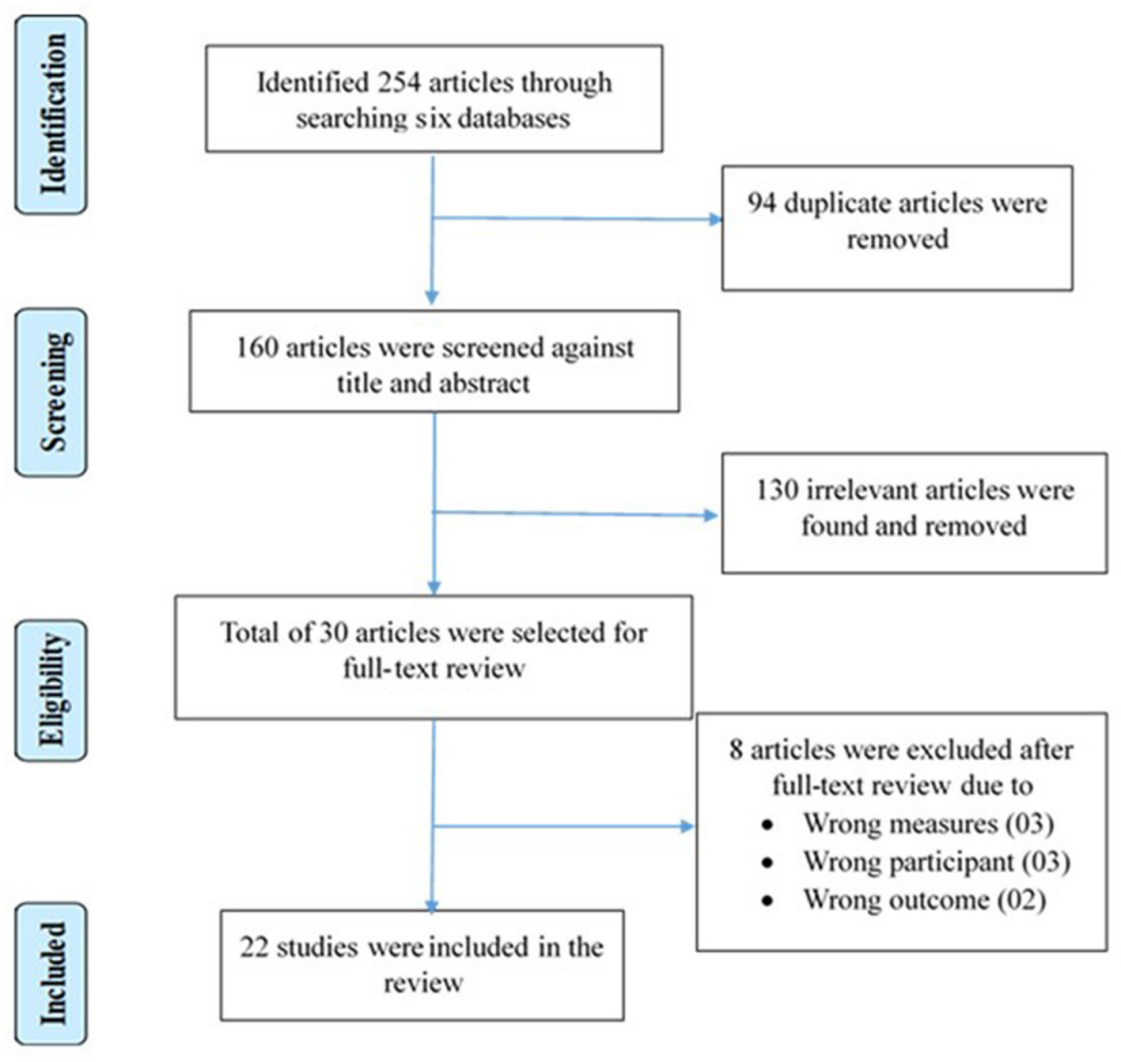
PRISMA flowchart of the article selection process.

Article selection occurred in four stages. In the first stage, 254 articles were identified from six databases with predetermined keywords. In the second stage, 94 duplicates were first excluded, and then the remaining 160 articles were screened based on their titles and abstracts, leaving 30 articles for full-text review. The abstracts of the articles that did not match the inclusion criteria were excluded at this stage. The third stage concerned checking the eligibility criteria of these 30 articles. Finally, 22 articles meeting the criteria were selected for inclusion in this review. PRISMA Statement guidelines (50) were followed throughout this process. Covidence (www.covidence.org) software was used to screen and select articles systematically. To minimize bias, the first author and an independent assessor conducted the entire process separately, after which a meeting was arranged to resolve any discrepancies in the article selection. Finally, 22 articles reporting ten quantitative, nine qualitative, and three mixed-method studies were selected for the current review. The mixed-method studies were the ones that adopted both qualitative and quantitative approaches in data collection and processing. Figure 1 displays the PRISMA flowchart for the article selection process.

### Data Extraction

The first author extracted all key data, such as samples' demographic details, study information, measures, and findings. For accuracy, the third author cross-checked all information. Upon agreement of both authors, the data were finalized before synthesis. The entire data extraction process was completed using NVivo-12 (51).

### Quality Appraisal

Study quality was assessed with the Mixed Method Appraisal Tool (MMAT) (52), demonstrated to be a valid and reliable instrument (53, 54). The MMAT can evaluate qualitative, quantitative, and mixed-method studies. There is a compulsory screening section to ensure that the eligibility criteria are met. The evaluation of quantitative studies includes three separate criteria for randomized controlled trials, comparative studies, and descriptive studies. There are different sets of questionnaires for qualitative and mixed-method studies. An overall scoring system is excluded from the updated version of MMAT (52). Instead, a detailed investigation of the articles for each criterion, based on evaluating the strength of methodological features, is suggested.

### Data Synthesis

**Figure 2:**
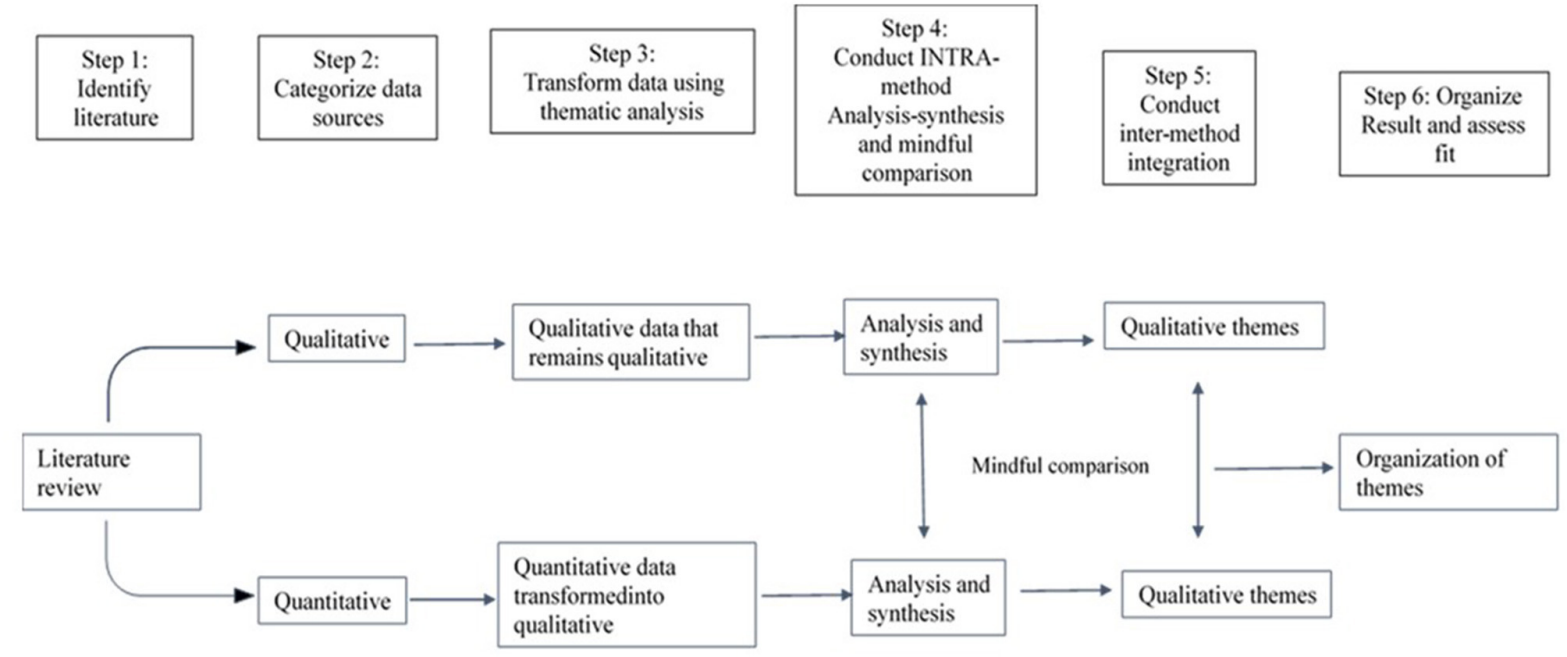
Steps of basic convergent qualitative meta integration.

This systematic review includes articles with quantitative, qualitative, and mixed-method study designs. Therefore, the basic convergent qualitative meta-integration technique, with six steps for synthesizing data, was adopted—see Figure 2 (55). This procedure is used to combine qualitative, quantitative, and mixed-method study findings in a systematic review paper. After categorizing different data sets, all quantitative findings are converted to qualitative data using thematic coding. All coded data are then synthesized and integrated based on the theme.

## Results

### Appraisal of the Studies

All 22 articles selected for the current review satisfied MMAT screening requirements (Supplementary Table 2). The nine qualitative articles met all five criteria listed in the qualitative evaluation section of the MMAT. Out of ten quantitative articles, two adopted non-randomized designs, and eight used descriptive designs. The two articles with non-randomized study designs met all the relevant criteria (56, 57). Six out of eight quantitative studies using descriptive designs neither indicated the sampling strategy nor recruited samples that were representative of the population (24, 28, 58–61). One mixed-method study (59) had a quantitative description design; however, it did not meet two criteria related to sampling strategy and representativeness. All mixed-method studies met full MMAT criteria for qualitative and mixed-method studies (57, 59, 62).

### Description of Studies

A total of 1,222 refugee and asylum-seeking participants, 759 adults and 463 children, took part in the reviewed studies. Sixteen studies involved only adults, four studies only children, and two studies included both children and adults of various nationalities and ethnicities (Table 1). A range of different tools was used in the quantitative studies to assess PTSD, traumatic events, the impact of trauma, depression, and individual's strengths and difficulties (Table 1). In the qualitative studies, participants' psychological reactions were extracted from the life narratives they produced.

**Table 1:** Summary of the articles selected for the review.

**Article**	**Type of study**	**Setting**	**Sample size**	**Age**	**Gender**	**Nationality/Ethnicity**	**Autobiographical memory measures**	**Mental health outcome measures**
Hollo (63)	Qualitative	Germany	3 refugees	Not mentioned	All females	Estonia	Autobiographical narrative	Autobiographical narrative
Ramsgaard and Bohn (64)	Quantitative	Denmark	34 children of refugee parents with PTSD and 34 matched community control group	10–18 years	14 boys and 20 girls	Somalia (*n* = 13), Iraq (*n* = 6), Kosovo (*n* = 4), Afghanistan (*n* = 3), Kuwait (*n* = 3), Palestine (*n* = 3), and Bosnia (*n* = 2)	Asked to write down past and future life events. They were also asked to write about an AM event that happened last weekend	Past and future life events were coded for overall emotional tone on the 5-point Likert scale
Chen and Schweitzer (65)	Qualitative	Australia	30 students and 5 parents	Students' age 11–18, parents' unknown	9 female and 21 males	Albanian, Congolese, Eritrean, Ethiopian, Indonesian, Iranian, Iraqi, Karen, Samoan, Somali, Sudanese, Syrian, Thai, Vietnamese, Bayat/Hazara/Afghan, Albanian, Chinese	Narrative storytelling	Narrative storytelling
Wildschut et al. (56)	Quantitative	Saudi Arabia	190 refugees	18–64 years	116 men, 74 women	Syrian	Recalling nostalgic or ordinary memory from their past with rating the memory between positive affect and negative affect	Psychological functions were assessed by four questionnaires with a 6-point rating scale Wagnild and Young's (66) 25-item Resilience Scale (RS) with a 7-point rating scale
Petta et al. (58)	Quantitative	Rome	14 refugees	20–37 years	All males	African	Lifespan Memory Interview in the Asylum Seekers (LMI-AS)	Two questions were asked to assess the level of stress and it was rated by VAS
Békés et al. (59)	Mixed	Hungary	20 refugees	Adult	6 males, 14 females	Jewish from Hungary and neighboring countries	In-depth interview	In-depth interview
Reebs et al. (61)	Quantitative	Israel	110 men	Adult Mean age 32.7, SD 6.49	All male participants	Sudanese refugees	Instructed to recall sad or fearful experiences by choosing self-immersed or self-distanced approaches	Mollica et al. (67) Emotional reactivity was measured by asking two questions with 5-point ratings
Wittekind et al. (68)	Quantitative	Germany	44 displaced people and 23 non-displaced, non-traumatized individuals	Elderly people born between 1932 and 1941	All male participants	From former parts of Germany (e.g., Silesia, Prussia)	AMT: Cue word presented on a card with 3 practice word sessions. 15 words (5 positive, 5 negative, 5 neutral) were presented, and they were asked to generate a specific memory. They were aided with prompt questions until the time limit expired	Post-traumatic Diagnostic Scale (PDS) (69): Diagnostic questionnaire for PTSD with severity assessment Hamilton Depression Scale (HDRS) (70), Vocabulary test using Intelligence test (Mehrfachwahl-Wortschatz-intelligenztest-B) (71)
Kevers et al. (72)	Qualitative	Belgium	Five families with 2–5 children	Adult parents and children aged at least 12 years	Not mentioned	Kurdish	Semi-structured Narrative interview	Narratives of interview
Pineteh and Mulu (73)	Qualitative	South Africa	20 participants	Age range 25–55 years	Female 6 Male 14	Cameroonians	Narrative interview	Narrative of interview
Huemer et al. (57)	Mixed	Austria	28 participants and a control group of 168 students.	Age between 15 and 18 years Control group: 14–18 (*M* = 16, *SD* = 1.3)		From African countries, including Gambia, Somalia, Nigeria, Kenya, Ghana, and Eritrea	Asked to remember a stressful event or random memory	Referential activity for measuring emotional reaction using the narratives
Panter-Brick et al. (9)	Quantitative	Afghanistan and Pakistan	331 students.	11–16 years old students Mean age 13.23 years	In Afghanistan Female 119 Male 115 In Pakistan Female 42 Male 55	Afghans	Open space for mentioning the most distressing traumatic events	Traumatic Event Checklist Child revised impact of events scale Depression self-rating scale (74) Strength and weakness scale Self-reported questionnaire for caregivers
Skardalsmo Bjorgo and Jensen (75)	Qualitative	Norway	34 unaccompanied adolescents	13–19 years old (*M* = 16.4, *SD* = 1.5)	Female 4 Male 30	From 8 different countries; mostly in Afghanistan (56%), Eritrea (18%), and Sri Lanka (9%)	Semi-structured narrative interview with contained upbringing, past life, and future life	Emotional reaction during an interview
Wylegała (76)	Qualitative	Krzyż /Zhovkva	33 biographical interviews	People who were 3–15 years old during migration	Not mentioned	Polish and Ukrainian	Biography	Narratives of biography
Graham et al. (8)	Quantitative	UK	38 people	Adult	Female 11 Male 27	African, South American, Middle Eastern, and Eastern European	Autobiographical Memory Test [AMT; (77)] with 16 emotion-related cue words	SCID-IV for diagnostic confirmation of PTSD (78)
Krahn (79)	Qualitative	Canada	16 refugees	Survivors were 78–96 years old, while adult children were 51–67	All females	Russian	Participant observation, interviewing, and archival research	Participant observation, interviewing, and archival research
Nets-Zehngut (80)	Qualitative	Palestine	4 oral history projects including interviews of 131 refugees The historical document of Israeli history was written by a famous history writer	Adult	Not mentioned	From 38 different localities of Palestine	Oral history/narrative Secondary sources of Israeli documented history Studies of Palestinians	Oral history/narrative interview Secondary sources of Israeli documented history Studies of Palestinians
Moradi et al. (24) (Study 1)	Quantitative	UK	37 people	Adult Mean age 38.5 years and SD 14.72	Female 18 Male 19	24 Kosovan Albanians and 13 Bosnians	AMT:10 emotion-related cue words (5 positive and 5 negative) were given	Post-traumatic Diagnostic Scale (PDS) (81): Diagnostic questionnaire for PTSD with severity assessment. The translated version was used
Eytan et al. (60)	Quantitative	Switzerland	7 refugees	18–50 years old Mean 26.85 years	Not mentioned	Sub-Saharan West Africans	Asked to recall any traumatic memory and happy memory in two different sessions with a 6-week interval.	Impact of Event Scale (82) Hospital Anxiety and Depression Scale (HADS) (83)
Herlihy et al. (28)	Quantitative	England 1999 and 2000	39 refugees	The range between 18 and 64 Mean 39.5 and SD 14.5	Female 20 Male 23	27 Kosovan Albanians and 16 Bosnians	Asked to recall a traumatic and a non-traumatic event from their lives. Then they were presented with 15 questions to evaluate the events Rated the events as peripheral or central to their experience The interview was taken twice with several weeks' (3–32) interval	Post-traumatic Diagnostic Scale (PDS) (81): Diagnostic questionnaire for PTSD with severity assessment Beck Depression Invention-II (84)
Miller et al. (85)	Qualitative	Chicago	28 adult Bosnian refugees	Adult age Mean 49.37 years and SD 10.02	Female 18 Male 10	Bosnian refugees	Semi-structured narrative interview: Refugee Distress and coping Interview (RDCI) was developed and used to explore pre-peri-post-exile stressors	Refugee Distress and coping Interview (RDCI) was used to assess distress
Weine et al. (62)	Mixed	USA	20 refugees including 6 adolescents	Age range from 13 to 62	Female 10 Male 10	Bosnian	Narrative Interview: asked about themselves, families, and events during the genocide	Assessment of psychiatric history Mental state examination PTSD symptom scale Communal Traumatic Experiences Inventory Global Assessment of Functioning Scale, SCL-90-R (86)

### Themes Extracted

Four recurrent themes were extracted: (1) memory activation methods, (2) memory features, (3) memory content, and (4) refugee people's mental health. Theme 1 addresses the first research question, themes 2 and 3 cover the second research question and theme 4 focuses on the third research question.

#### Memory Activation Methods

Our first research question was to identify the common research methods used in autobiographical memory and mental health research on refugee and asylum-seeking people. We found that different methods were used in the selected studies. Narrative interviews were the most frequently used method; twelve out of 22 studies utilizing this method (58, 59, 62, 63, 65, 72, 73, 75, 76, 79, 80, 85). In these studies, participants were asked to recall the days before migration, and describe the genocide or conflict they witnessed during their migration journey and/or after resettling in the host country. Two of these studies used the archived data in which narrative interviews of refugee participants were preserved (76, 80). Nets-Zehngut (80) compared archival narratives collected from 131 Palestinian refugee people with the recorded history of the Palestinian refugee people's lives.

The second most common method used was the important memory method. Seven studies utilized this method in which participants were asked to report their most traumatic or distressful life events (9, 28, 56, 57, 60, 61, 64). In these studies, participants mostly recalled negative life events with the occasional retrieval of happy, neutral, nostalgic, or random memories. Participants of two studies were asked to imagine future events along with the retrieval of autobiographical memories (64, 75). In two studies (28, 60), the consistency of important memories was assessed through repeated recall. Lastly, the cue word method was utilized in three studies (8, 24, 68) in which different combinations of common words were used.

#### Memory Features

Three different memory features were extracted: (1) retrieval failure, (2) inconsistent memory, and (3) reduced memory specificity. This theme partly addresses our second research question.

##### Retrieval Failure

There were four studies (9, 58, 59, 62) in which both adolescents and elderly participants appeared to be unable to recall any memory they reported previously, particularly traumatic, and stressful memories. In general, refugee people were less likely to recall self-distant memories compared to self-related memories (61). Sometimes, trauma-exposed refugee participants expressed the emotional content of the events rather than the details of the events (59).

##### Inconsistent Memory

A common feature of refugee people's autobiographical memory was that they lacked consistency. Three studies (9, 28, 58) revealed that participants were unable to describe the same memory that they had reported several weeks ago. The inconsistency was more apparent for the marginal description of an event than for the description of the event core (28). Adolescents were more likely than adults to alter their distressful life experiences (57); however, their simulation of future events was coherent with their memories (64). Inconsistencies between oral histories and academically documented stories were also observed (80), with oral histories being more amorphous and variant. Nets-Zehngut (80) found high discrepancies among memories of specific events recalled by people from the same community but living in different locations. Only one study found mostly identical memory protocols produced six weeks apart (60).

##### Reduced Memory Specificity

Memory specificity was investigated in three studies (8, 24, 68). Two studies revealed that specificity was higher for non-traumatic memories than for traumatic memories (8, 68). However, the proportion of specific memories recalled did not vary between everyday negative events and major traumatic memories (68). One refugee woman's narrative indicated she might have images of the traumatic events, but she did not give specific details when recalling the events (63). Graham et al. (8) demonstrated that recall of specific autobiographical memory was less frequent among refugee people with PTSD. Wittekind et al. (68) revealed that positive or non-traumatic cues elicited more specific memories than negative or trauma-related cues. Refugee participants with depressive symptoms were found to produce less specific autobiographical memories compared to refugee participants who were not depressed (8). Moradi et al. (24) revealed that the specificity of refugee people's memories was associated with core symptoms (e.g., flashbacks, avoidance) of PTSD. They found autobiographical memories to be negatively linked to re-experiencing the trauma, yet positively associated with avoidance behavior (24).

#### Memory Content

Nine different types of memory content were extracted from autobiographical memories produced by the participants in the analyzed studies. This theme also addresses our second research question.

##### Changing Status and Identity Confusion

In six studies (64, 72, 73, 76, 79, 85), both adolescent and adult refugee participants mentioned an alteration of past identities and accountabilities. They perceived their refugee status as worthless compared to their pre-migration life (85). Their refugee status and future uncertainty affected adolescents' daily lives, cultural lives, and self-identities; thus, they tended to generate less normative cultural life scripts than non-refugee adolescents (64). Even refugee children felt inferior to their non-refugee peers (76). Pineteh and Mulu (73) reported that many refugee people felt humiliated and neglected while trying to find a job, sometimes hiding their refugee identity and avoiding their community. This trend was more common among children than adult participants (76). However, some adult refugee people presented themselves as protagonists to protect their original identities (73), and sometimes showed pride in their national identities (76). Kevers et al. (72) observed that Kurdish refugee participants believed that the rest of the world had been harboring a negative image of them; therefore, they tried to convince others to renew respect for their community. In their effort to secure self-identity, older refugee women preferred to talk about the positive sides of their life experiences (79).

##### Feeling of Loss

While talking about self, both older and younger refugee participants remembered their past lives and recounted what they had and what they had lost (63, 65, 72). Senior refugee people missed their home countries so much that they recounted many personal events even half a century after their migration (63). The sense of loss varied depending on the age of participants. Adults wished to get back their homes (72), whereas children focused more on their present lives and on reconstructing their identities in their new settlements (76).

##### Sense of Insecurity and Injustice

Feelings of insecurity are common among refugee people, even after resettling in a new country. An adult refugee woman narrated her constant childhood fear that she would lose her mother and grandmother during the war and resulting migration (63). Wylegała (76) found that children who were refugees reported they could not rely on their parents or elders, whom they had previously trusted, for safety. A refugee woman felt confused when her mother beat her brutally after being raped by a soldier at a very early age (63). Kevers et al. (72) found that maintaining silence was common among refugee family members when they felt unsafe or did not want to recall traumatic events as a kind of protective self-defense mechanism.

##### Social Isolation

After migration, refugee people felt isolated from society and discussed lost social bonds after migration that they were unable to recreate in their new environments (85). Adults who are refugees sometimes refused to meet other community members because meeting with their people might remind them of what they had lost. Children also felt isolated, neglected, and sometimes were bullied at school in the host country (63). However, these kinds of experiences were often attributed to an unfamiliar culture and language rather than to refugee status (76). Some refugee people also shared positive experiences; for example, they indicated they were often supported and appreciated by the local people (63).

##### Worth of Living

Older refugee participants were in a crisis concerning their perceptions of the meaning of life more so than younger refugee participants (85). Older refugee participants expressed some kind of purpose or aim in their pre-migration life narratives, but this purpose was utterly absent in their post-migration life narratives. They reported perceiving their looming death since they were exposed to war or conflict, viewing their death as happening only in a matter of time (63). Participants from three studies stated they felt disheartened when attempting to face the ultimate meaning of life (62, 72, 85). One participant from Weine et al. (62) expressed his disgrace and suffering by saying that sometimes he feels not human, but beast-like. However, refugee people with high resiliency reported high self-esteem, a belief in a positive meaning of life, and social attachment while recalling their nostalgic memories more so than refugee people with lower reported resiliency (56).

##### Lack of Resources for Adaptation

In three studies, participants recalled feeling added stress from a lack of resources for adapting to their new settings (63, 76, 85). According to Miller et al. (85), the difficulties faced by adult refugee people in gaining new practical knowledge, especially a new language, could intensify their feelings of incompetence and anxiety. According to Wylegała (76), adults tried to rationalize their current refugee status in a way that suggests their sacrifice could bring back their lost identities. They also iterated that their suffering and sacrifices symbolized their patriotism.

##### Promoting Own Culture

Participants of three studies introduced their own culture, norms, and values to the researchers (65, 72, 76). Adult—but not young—refugee participants appeared to enjoy talking about their home countries, customs, and cultures. They also continued to practice their traditions in their new settings (76). Chen and Schweitzer (65) found that adolescents honored their customs and heritage as a significant part of their identities.

##### Optimism

Albeit having multiple adverse life experiences, many refugee participants keep hope for change in their lives. This was observed in the narratives produced by participants in five studies (59, 64, 73, 76, 85). However, the tendency to be courageous and resilient was more visible in children and young adults than in elderly refugees (85). One study revealed that some refugee people had high hopes they would return to their ancestral homes one day (76). While simulating future life stories, both adolescents and adult refugees tended to be altruistic to their people (59, 64). Among refugee participants, optimism was identified as a protective factor (73) and a healthy coping strategy in a changed and challenging world (85).

##### Trauma Memories

Seven studies revealed that participants from refugee communities were more likely to talk about adverse life events they experienced during pre-migration violence, wartime conflict, and their migration journeys (57, 59, 62, 63, 68, 73, 75). Younger refugee participants were prone to recall violent events occurring at home or school (75). These negative memories were produced during interviews and laboratory conditions (68). Young refugee participants spontaneously recalled more traumatic memories than the control group (57) and the non-clinical population (68), even when they were not exposed to the events directly but only learned from others' narratives (63). In one study, adult participants described mental images of past traumatic events (e.g., murder, torture, and destruction) so vividly that they felt they were living with those images (62). However, participants from another study recalled the cognitive elements of traumatic events as if they were emotionally detached from those events (59). Sometimes, participants consciously avoided describing memories despite having clear images of traumatic events (63).

When the autobiographical memory of clinical populations (e.g., people with PTSD) was investigated, it was found that participants retrieved traumatic memories quite clearly regardless of the cues used to induce them. Wittekind et al. (68) claimed that PTSD-affected individuals can recall more traumatic events than those that are non-traumatized and those not affected by PTSD. Refugee participants' life narratives in two studies illustrated they could recall the adverse events that happened during their forced migration (73, 76). Participants described the journey as terribly long in extreme weather conditions and without a clear destination (63, 76). It was an unforgettable journey fraught with challenges and uncertainty for many refugee people, and at times they thought they might not be able to complete or survive it (63, 73).

#### The Mental Health of Refugee and Asylum-Seeking People

This theme addresses our third research question. Across the reviewed articles, PTSD, depression, negative reactions, and contradictory feelings were associated with refugee people's autobiographical memory. Three subthemes were extracted: (1) the presence of psychological disorders and symptoms, (2) negative emotions, and (3) contradictory feelings about experienced trauma.

##### Psychological Disorders and Symptoms

Many participants in eleven studies either suffered from PTSD or demonstrated symptoms of PTSD or post-traumatic distress (8, 9, 24, 28, 60–63, 68, 76, 79). Participants from all but two of those studies were found to be suffering from PTSD (9, 60). The remaining two studies investigated participants' traumatic life experiences and demonstrated how the trauma affected their mental health. Three studies examined the link between PTSD and autobiographical memory specificity (8, 24, 68). Autobiographical memory specificity was found to be differently associated with the four core PTSD symptoms: flashbacks, re-experiencing, avoidance, and hyperarousal; the more intense the flashback of a traumatic event, the lower the specificity of autobiographical memory (24). Another study revealed that re-experiencing and avoidance were more frequent than symptoms of hyperarousal among older refugee participants (62). Children particularly reported suffering from nightmares, hyper-arousal, and sleep disturbances (63, 76). However, Reebs et al. (61) found that post-traumatic stress symptoms did not predict avoidance behavior among refugee participants.

The consistency of recalling distressing autobiographical memory by refugee participants with chronic post-traumatic distress was quite low (9). Herlihy et al. (28) reported that the more severe the PTSD, the lower the consistency in refugee participants' memories. Depression was found to be a comorbid disorder for refugee participants suffering from PTSD (8, 79). As depression increased, autobiographical memory specificity was also reduced (68). Wide-ranging experiences, behaviors, and attitudes that may also reflect depression, such as bereavement, undermining behaviors, being offended, and in extreme cases, schizophrenic symptoms, were detected in refugee participants' narratives (62, 79).

##### Negative Emotional Reactions

Refugee people participating in nine studies expressed different types of negative emotional reactions: sadness, aggression, helplessness, and hopelessness concerning what they had lost, and a feeling of apprehension and distress regarding ongoing problems (59, 61–63, 65, 75, 76, 79, 85). Although adult refugee participants indicated that their children did not understand what was happening around them, negative emotions were frequently expressed in the narratives of child refugee participants (63, 65, 76). Some older participants reported occasionally feeling so mean that it was as if they did not have a soul (62).

##### Contradictory Feelings

Four studies noted the presence of contradictory feelings among adolescent refugee participants and observed adult refugees describing their childhood memories about traumatic events (63, 64, 75, 76). Along with negative moods, they expressed mixed feelings toward those who tortured them (75). When adult participants were children, they found war events were sometimes quite exciting and novel (63). This was evident when they were talking about the violence they experienced at home and/or school before migration (75). Interestingly, adolescents who were tortured during their childhood by parents or teachers missed being with or seeing them simply because they felt they were tortured due to their misbehavior or for their well-being. In one study, adult refugee participants reported they felt confused about the social and political conflict that took place during their young age because they were not mature enough to understand all the complexities involved (76). Adults also sometimes expressed mixed feelings while recalling nostalgic memories (56).

## Discussion

In this paper, we summarise the results of research investigating autobiographical memory and the mental health of refugee and asylum-seeking people recruited from different parts of the world. We had three broad research questions. First, we wanted to see what kind of methods were used to induce autobiographical memories; second, examine the features and content of autobiographical memories produced by the study participants; and third, explore mental health issues reported by the participants. The convergent meta-integration technique extracted four recurrent themes: (1) memory activation methods, (2) memory features, (3) memory content, and (4) mental health of the refugee and asylum-seeking people. The first theme addresses the first research question, the second and third themes are aligned with the second research question, and the fourth theme focuses on the third research question.

Concerning the first research question, most research has conducted narrative interviews to gather autobiographical memories (58, 59, 62, 63, 65, 72, 73, 75, 76, 79, 80, 85), followed by the important memory method, in which participants recalled traumatic and distressful life events (9, 28, 56, 57, 60, 61, 64), and the cued recall paradigm, in which memories were elicited by word cues (8, 24, 68). Most researchers preferred narrative interviews over other methods as this technique helped them collect an individual's coherent life stories rather than scattered life events (72). It is narrative stories through which one can better understand the types of trauma an individual experienced at different legs of life (72). Another advantage of narrative stories is that they often contain a participant's thoughts about the future including their wishes and dreams (59, 64, 73, 76, 85).

Focusing the second research question—the features of autobiographical memory, we observed that participants' memories were often inconsistent; descriptions of the same event were different at different points in time (9, 28, 57, 58). This pattern was similar across younger and older participants. They were also unable to recall many of their life events. They frequently recalled traumatic and distressful events without much detail, thus producing overgeneral memories (8, 24, 68). Older adults recalled more overgeneral memories than adolescents. However, several studies have shown that memories attached to negative emotions were more vivid and accurate than memories associated with positive emotions (59, 62, 68, 73, 75, 76). Inconsistent memory in both adults and children could be due to the bias of tunnel memory and observer memory (33). According to Rubin et al. (33), traumatic memory can be biased through (1) tunnel memory (i.e., a tendency to focus on memories that involved emotional provocation), (2) observer memory (i.e., recalling memories as if only an observer and not an active participant in the memory), and (3) personal goals and attitudes (i.e., referring to the personal or cultural significance of a memory).

Adult participants were more likely to focus on the core of negative life events; therefore, outer details of their memories were inconsistent (28), demonstrating tunnel memory bias (33). The tendency to share distressful memories could be a product of tunnel memory; yet, it could also be a useful mechanism for ameliorating negative emotions (87). Unlike adults, young participants either altered their memories or described without sufficient detail and affect. They also tended to see their traumatic experiences from the view of an observer, thus avoiding any emotional attachment with the event (57, 63). It is worth noting that not all participants in the reviewed studies were patients with PTSD, although Rubin et al. (33) claimed memory biases are particularly related to PTSD symptoms.

Overgeneral memory (OGM), or a lack of specificity in autobiographical memory, was quite common among refugee participants. Conway and Pleydell-Pearce (19) suggested aborted generative search within the memory hierarchy as the reason for OGM (19, 88). OGM was explained further by Williams' (30) CaR-FA-X model, which describes three mechanisms: Capture and Rumination (CaR), Functional Avoidance (FA), and lack of executive control (X). OGM happens when generative search in the memory hierarchy becomes stuck in the intermediate level (Capture) and starts ruminating with the memory (Rumination). People ruminate more when the memory, or the cue, is linked with self (89). FA works as a gateway to block the negative emotional memory from coming out (30); therefore, people avoid details of the negative memory by providing a general response to the memory. It increases the tendency to talk more about the negative event in a generic manner by people who have experienced trauma (90, 91). Both adult and younger refugee participants showed similar tendencies when sharing their distressful experiences. Additionally, a lack of cognitive control may make the generative retrieval process slower and produce fewer specific memories.

While examining memory contents (part of the second research question), various psychosocial features were extracted—changing status and identity confusion, feelings of loss, a sense of insecurity and injustice, and social isolation. These results can be explained using the ADAPT model (92), which focuses on refugee people's experiences of psychosocial issues. The model proposes five psychosocial pillars that are required for the stability of society and the mental health of its people. The core pillars are safety and security, social bonding, role and responsibilities, justice, and the meaning of life. In the refugee community, the pillars are likely to be disrupted due to war, conflict, and forced displacement. Silove (92) asserts that the prolonged collective victimization which has created this disruption is likely to result in poor mental health.

The content extracted from participants' narratives frequently reflected the disruption of core psychosocial pillars of the ADAPT model. Participants talked about their changed status and loss of identity, insecurities, and injustices, and loss of attachment and social bonding (92). There were notable similarities between the memory content produced by children and adult participants. Like most adults, children also felt unsafe, even when they were with their parents and close relatives. They felt inferior and socially isolated, and they hid their refugee identity before non-refugee peers. As indicated in their narratives, adults occasionally avoided the company of their community members and felt the loss of attachment to their previous lives. Some also wished to return to their home country. However, young participants were more resilient, focused on their present lives, and hopeful about their futures.

There were several studies in which participants gave vivid descriptions of traumatic events (59, 62, 68, 73, 75, 76). This result opposes the mainstream literature in which a link between trauma and OGM has been established. To make sense of this unexpected result, we should take into account the practical aspects of day-to-day survival needs for refugee people. To establish asylum-seeking credibility to legal authorities, refugee people must repeatedly recall and report their adverse, life-threatening experiences in their countries before migration and during migration journeys (93). They are required to narrate the central details of those traumatic events over and over again to substantiate their claim for refugee status (48). As the recollection of those events was essential for defending their refugee identities, they recalled them without much anguish. The memories' ability to create distress was dissipated because their detailed recollection was likely to bring needed material and psychological resources to refugee people.

With regards to the third research question, the refugee people exhibited a wide range of mental health issues, ranging from experiencing sadness and anxiety to being diagnosed with psychiatric disorders, such as PTSD and depression (61, 75, 85). Among psychiatric disorders, PTSD and depression have been frequently studied in refugee people as comorbid conditions (8, 9, 62, 68). Symptoms of PTSD were associated with reduced memory specificity. Refugee participants with PTSD were more inconsistent in the repeated recall of their life stories than non-clinical refugee participants (9, 28). Refugee participants, while discussing their past lives, expressed sadness, hopelessness, helplessness, anger, feelings of being lost, anxiety, and distress. For the most part, adolescent refugee participants expressed conflicted feelings. Impaired mental health among refugee people has been attributed to different competing theories; while some theories emphasize the loss of resources due to forceful eviction (94), others highlight the challenges refugee people normally encounter in their post-migration living conditions (15).

According to the conservation of resources theory (94), the loss of resources in predetermined domains, such as personal, familial, or social, ultimately disrupts an individual's self-control and resilience. In this review, we noted frequent mention of the deprivation of adequate resources in life narratives (63–65, 72, 73, 76, 79, 85). Therefore, a loss of resources may be the reason for existing psychological distress among refugee people. In contrast, the ecological theory suggests that adverse post-migration living conditions determine refugee people's mental health (15). After migration, they confront many daily living stressors, such as unsafe living conditions, inadequate survival resources, isolation, lack of employment opportunities, and many more. Evidence demonstrates that the stressful post-conflict situation mediates post-trauma-related mental health (95). In the present review, issues related to stressful post-migration conditions, for instance, social isolation and job crisis in an unsupported and unsafe environment, repeatedly appeared in the narratives produced by the participants (63, 73, 76, 85).

Several articles in this systematic review have shown a link between trauma-induced disorders (e.g., PTSD and depression) and autobiographical memory impairment. Some researchers have claimed that impairments of war-trauma memories could be recovered over time (96), but that traces of PTSD could remain for a long time. Therefore, it is still inconclusive what type of memories were more likely to be impaired among refugee people. The debate remains for what contributes more to the disruption of refugee mental health. Is it trauma exposure or ongoing hazards and living conditions after migration, or both?

### Limitations

We have encountered several challenges while conducting this systematic review. A major challenge was to compile results appearing in a diverse set of studies, as the sample size, age, nationality, and ethnicity of participants varied significantly. The study designs, measurement tools, and assessment methods were also quite diverse. Particularly in the qualitative studies, the methods used to assess memory narratives did not follow standard procedures for measuring autobiographical memory. We included these studies because they investigated memory components that are of particular importance for studying refugee memory. Due to a lack of resources, we did not include grey literature or articles published in languages other than English. Besides, the quality assessment tool required subjective justification.

### Conclusion

This review offers a comprehensive summary of trauma, autobiographical memory, and mental health of refugee and asylum-seeking people. Researchers mostly used narrative interviews, the important memory method, and cued recall paradigm to activate autobiographical memories—in some instances with the instruction to recall traumatic and emotional life experiences. The recall of autobiographical memory by refugee people was often disrupted; they faced difficulty in both describing their life experiences in detail and describing the same memory at a later point—sometimes simply being unable to recall it. However, quite unexpectedly, in some studies, refugee participants were able to recall traumatic life experiences in sufficient detail. As it was required for them to vividly recall these memories in front of legal authority to defend their refugee status, they did it without cutting their description short while minimizing their distress. It appears that the need for providing detailed recollections of distressful life experiences outweighed the loss of re-experiencing those unpleasant events. The memories produced by refugee people frequently delineated their loss of physical and psychological resources and the challenges they faced in their resettlement environment. PTSD, depression, and feelings of hopelessness were common among refugee people. We recommend that future research should focus on the vulnerability of trauma and non-trauma memories at different points after trauma exposure to ascertain how they contribute to the impaired mental health of refugee people. These findings could be useful for researchers and professionals interested in working with trauma memory and refugee mental health.

## Data Availability Statement

The original contributions presented in the study are included in the article/Supplementary Material, further inquiries can be directed to the corresponding author.

## Author Contributions

SH and SK conceived the research idea. SK was involved in data extraction, formal analysis, and written the original draft. The manuscript was finally reviewed and edited by SKK and SH. The project was supervised by SH. All authors contributed to the article and approved the submitted version.

## Conflict of Interest

The authors declare that the research was conducted in the absence of any commercial or financial relationships that could be construed as a potential conflict of interest.
