# Supplementary material for: A Systematic Review of Autobiographical Memory and Mental Health Research on Refugees and Asylum Seekers
Source: Front Psychiatry. 2021 Jun 4;12:658700. doi: 10.3389/fpsyt.2021.658700 (PMC8211731; doi:10.3389/fpsyt.2021.658700)
Supplement: Supplementary file 1 [file Data_Sheet_1.pdf]

## Supplementary Tables

**S1 Table. Number of articles found in different databases using the specific search string**

| Search string | Database | Results found |
|---|---|---|
| refuge* OR "asylum seeker*" OR displace* OR "refuge* camp*" OR diaspora*<br>AND<br>"autobiograph* memor*" OR "life script*" OR "life narrative*" OR "overgeneral* memor*" OR "memory specific*" OR "extended memor*" OR "episod* memor*"<br>AND<br>"mental health" OR "mental disorder*" OR "psychological disorder*" OR "psychiatric* disorder*" OR psychosocial OR psychiatr* OR wellbeing OR distress OR trauma OR psychol* OR functioning OR depressi* OR anxiety OR ptsd OR "post-traumatic stress disorder*" | EBSCOhost | 49 |
| | PILOTS | 11 |
| | PsycINFO | 38 |
| | PubMed | 51 |
| | Scopus | 45 |
| | Web of Science | 60 |

**S2 Table.** **Appraisal of the articles using MMAT**

| Types of studies<br>Screening | Methodological quality criteria (see tutorial for definitions and examples) | Hollo 2020 | Ramsgaard and Bohn 2019 | Chen and Schweitzer 2019 | Wildschut et al. et al., 2019 | Petta et al. 2018 | Békés et al. 2017 | Reebs 2017 | Wittekind 2017 | Kevers 2017 | Pineteh 2016 | Huemer 2016 |
|---|---|---|---|---|---|---|---|---|---|---|---|---|
| | S1. Are there clear research questions? | Yes | Yes | Yes | Yes | Yes | Yes | Yes | Yes | Yes | Yes | Yes |
| | S2. Do the collected data allow to address the research questions? | Yes | Yes | Yes | Yes | Yes | Yes | Yes | Yes | Yes | Yes | Yes |
| **1. Qualitative** | 1.1. Is the qualitative approach appropriate to answer the research question? | Yes | N/A | Yes | N/A | N/A | Yes | N/A | N/A | Yes | Yes | Yes |
| | 1.2. Are the qualitative data collection methods adequate to address the research question? | Yes | N/A | Yes | N/A | N/A | Yes | N/A | N/A | Yes | Yes | Yes |
| | 1.3. Are the findings adequately derived from the data? | Yes | N/A | Yes | N/A | N/A | Yes | N/A | N/A | Yes | Yes | Yes |
| | 1.4. Is the interpretation of results sufficiently substantiated by data? | Yes | N/A | Yes | N/A | N/A | Yes | N/A | N/A | Yes | Yes | Yes |
| | 1.5. Is there coherence between qualitative data sources, collection, analysis and interpretation? | Yes | N/A | Yes | N/A | N/A | Yes | N/A | N/A | Yes | Yes | Yes |
| **3. Quantitative** | 3.1. Are the participants representative of the target population? | N/A | N/A | N/A | Yes | N/A | N/A | N/A | N/A | N/A | N/A | Yes |

| | | | | | | | | | | | | |
|---|---|---|---|---|---|---|---|---|---|---|---|---|
| **Non randomized** | 3.2. Are measurements appropriate regarding both the outcome and intervention (or exposure)? | N/A | N/A | N/A | Yes | N/A | N/A | N/A | N/A | N/A | N/A | Yes |
| | 3.3. Are there complete outcome data? | N/A | N/A | N/A | Yes | N/A | N/A | N/A | N/A | N/A | N/A | Yes |
| | 3.4. Are the confounders accounted for in the design and analysis? | N/A | N/A | N/A | Yes | N/A | N/A | N/A | N/A | N/A | N/A | Yes |
| | 3.5. During the study period, is the intervention administered (or exposure occurred) as intended? | N/A | N/A | N/A | Yes | N/A | N/A | N/A | N/A | N/A | N/A | Yes |
| **4. Quantitative descriptive** | 4.1. Is the sampling strategy relevant to address the research question? | N/A | Yes | N/A | N/A | No | No | No | Yes | N/A | N/A | N/A |
| | 4.2. Is the sample representative of the target population? | N/A | Yes | N/A | N/A | No | No | No | Yes | N/A | N/A | N/A |
| | 4.3. Are the measurements appropriate? | N/A | Yes | N/A | N/A | Yes | Yes | Yes | Yes | N/A | N/A | N/A |
| | 4.4. Is the risk of nonresponse bias low? | N/A | Yes | N/A | N/A | Yes | N/A | Yes | Yes | N/A | N/A | N/A |
| | 4.5. Is the statistical analysis appropriate to answer the research question? | N/A | Yes | N/A | N/A | Yes | Yes | Yes | Yes | N/A | N/A | N/A |
| **5. Mixed methods** | 5.1. Is there an adequate rationale for using a mixed methods design to address the research question? | N/A | N/A | N/A | N/A | N/A | Yes | N/A | N/A | N/A | N/A | Yes |
| | 5.2. Are the different components of the study effectively integrated to answer the research question? | N/A | N/A | N/A | N/A | N/A | Yes | N/A | N/A | N/A | N/A | Yes |

| | | | | | | | | | | | | |
|---|---|---|---|---|---|---|---|---|---|---|---|---|
| | 5.3. Are the outputs of the integration of qualitative and quantitative components adequately interpreted? | N/A | N/A | N/A | N/A | N/A | Yes | N/A | N/A | N/A | N/A | Yes |
| | 5.4. Are divergences and inconsistencies between quantitative and qualitative results adequately addressed? | N/A | N/A | N/A | N/A | N/A | Yes | N/A | N/A | N/A | N/A | Yes |
| | 5.5. Do the different components of the study adhere to the quality criteria of each tradition of the methods involved? | N/A | N/A | N/A | N/A | N/A | Yes | N/A | N/A | N/A | N/A | Yes |

## S2 Table. Appraisal of the articles using MMAT (Continuation)

| Types of studies | Methodological quality criteria (see tutorial for definitions and examples) | Graham 2014 | Moradi 2008 | Eytan 2008 | Herlihy 2002 | PanterBrick 2015 | Krahn 2013 | Miller 2002 | Skårdalsmo Bjørgo, 2015 | Wylegała 2015 | Nets-Zehngut 2011 | Weine 1995 |
|---|---|---|---|---|---|---|---|---|---|---|---|---|
| Screening | S1. Are there clear research questions? | Yes | Yes | Yes | Yes | Yes | Yes | Yes | Yes | Yes | Yes | Yes |
| | S2. Do the collected data allow to address the research questions? | Yes | Yes | Yes | Yes | Yes | Yes | Yes | Yes | Yes | Yes | Yes |
| **1. Qualitative** | 1.1. Is the qualitative approach appropriate to answer the research question? | N/A | N/A | N/A | N/A | N/A | Yes | Yes | Yes | Yes | Yes | Yes |
| | 1.2. Are the qualitative data collection methods adequate to address the research question? | N/A | N/A | N/A | N/A | N/A | Yes | Yes | Yes | Yes | Yes | Yes |
| | 1.3. Are the findings adequately derived from the data? | N/A | N/A | N/A | N/A | N/A | Yes | Yes | Yes | Yes | Yes | Yes |
| | 1.4. Is the interpretation of results sufficiently substantiated by data? | N/A | N/A | N/A | N/A | N/A | Yes | Yes | Yes | Yes | Yes | Yes |
| | 1.5. Is there coherence between qualitative data sources, collection, analysis and interpretation? | N/A | N/A | N/A | N/A | N/A | Yes | Yes | Yes | Yes | Yes | Yes |
| **3. Quantitative Non randomized** | 3.1. Are the participants representative of the target population? | N/A | N/A | N/A | N/A | N/A | N/A | N/A | N/A | N/A | N/A | N/A |
| | 3.2. Are measurements appropriate regarding both the outcome and intervention (or exposure)? | N/A | N/A | N/A | N/A | N/A | N/A | N/A | N/A | N/A | N/A | N/A |

| | | | | | | | | | | | | |
|---|---|---|---|---|---|---|---|---|---|---|---|---|
| | 3.3. Are there complete outcome data? | N/A | N/A | N/A | N/A | N/A | N/A | N/A | N/A | N/A | N/A | N/A |
| | 3.4. Are the confounders accounted for in the design and analysis? | N/A | N/A | N/A | N/A | N/A | N/A | N/A | N/A | N/A | N/A | N/A |
| | 3.5. During the study period, is the intervention administered (or exposure occurred) as intended? | N/A | N/A | N/A | N/A | N/A | N/A | N/A | N/A | N/A | N/A | N/A |
| **4. Quantitative descriptive** | 4.1. Is the sampling strategy relevant to address the research question? | Yes | No | No | No | Yes | N/A | N/A | N/A | N/A | N/A | No |
| | 4.2. Is the sample representative of the target population? | Yes | No | No | Yes | Yes | N/A | N/A | N/A | N/A | N/A | No |
| | 4.3. Are the measurements appropriate? | Yes | Yes | Yes | Yes | Yes | N/A | N/A | N/A | N/A | N/A | Yes |
| | 4.4. Is the risk of nonresponse bias low? | Yes | Yes | Yes | Yes | Yes | N/A | N/A | N/A | N/A | N/A | Yes |
| | 4.5. Is the statistical analysis appropriate to answer the research question? | Yes | Yes | Yes | Yes | Yes | N/A | N/A | N/A | N/A | N/A | Yes |
| **5. Mixed methods** | 5.1. Is there an adequate rationale for using a mixed methods design to address the research question? | N/A | N/A | N/A | N/A | N/A | N/A | N/A | N/A | N/A | N/A | Yes |
| | 5.2. Are the different components of the study effectively integrated to answer the research question? | N/A | N/A | N/A | N/A | N/A | N/A | N/A | N/A | N/A | N/A | Yes |
| | 5.3. Are the outputs of the integration of qualitative and quantitative components adequately interpreted? | N/A | N/A | N/A | N/A | N/A | N/A | N/A | N/A | N/A | N/A | Yes |

| | | | | | | | | | | | |
|---|---|---|---|---|---|---|---|---|---|---|---|
| 5.4. Are divergences and inconsistencies between quantitative and qualitative results adequately addressed? | N/A | N/A | N/A | N/A | N/A | N/A | N/A | N/A | N/A | N/A | Yes |
| 5.5. Do the different components of the study adhere to the quality criteria of each tradition of the methods involved? | N/A | N/A | N/A | N/A | N/A | N/A | N/A | N/A | N/A | N/A | Yes |
